# High-Risk HPV Persistence and Clearance Patterns Among Women in Ethiopia: A Longitudinal Study

**DOI:** 10.2147/IJWH.S544062

**Published:** 2025-10-22

**Authors:** Brhanu Teka, Adamu Addissie, Adane Mihret, Muluken Gizaw, Welelta Shiferaw, Zewditu Chanyalew, Andreas M Kaufmann, Eva Johanna Kantelhardt, Tamrat Abebe

**Affiliations:** 1 Department of Microbiology, Immunology and Parasitology, School of Biomedicine and Laboratory Sciences, College of Health Sciences, Addis Ababa University, Addis Ababa, Ethiopia; 2 Global Health Working Group, Martin-Luther-University, Halle-Wittenberg, Germany; 3 Department of Epidemiology and Biostatistics, School of Public Health, College of Health Sciences, Addis Ababa University, Addis Ababa, Ethiopia; 4 Armauer Hansen Research Institute, Addis Ababa, Ethiopia; 5 Department of Psychiatry, School of Medicine, College of Health Sciences, Addis Ababa University, Addis Ababa, Ethiopia; 6 Department of Pathology, St. Paul Hospital Millennium Medical College, Addis Ababa, Ethiopia; 7 Department of Gynecology, Charité–Universitätsmedizin Berlin, Berlin, Germany; 8 Institute for Medical Epidemiology, Biometrics and Informatics, Martin-Luther-University Halle-Wittenberg, Halle, Germany

**Keywords:** HPV, persistence, high-risk genotypes, natural history, cervical cancer, Ethiopia

## Abstract

**Purpose:**

High-risk human papillomavirus (hr-HPV) types are the primary cause of cervical and anogenital cancers. Understanding patterns of persistence, clearance, and re-infection is essential, as persistent infections contribute to precancerous and invasive lesions. This study evaluated hr-HPV infection dynamics and genotype specific outcomes over two years among Ethiopian women.

**Patients and Methods:**

A cohort of 893 women aged 30–49 years was followed for two years using self-collected samples for multiplexed hr-HPV genotyping by BSGP5+/6+ PCR. Women testing positive were re-evaluated at 6 and 24 months with HPV genotyping, Visual Inspection with Acetic Acid (VIA) and cytology. Persistent infection was defined as continuous HPV DNA presence over consecutive visits, while clearance indicated no detectable virus. Re-infection occurred if a cleared HPV type reappeared, or a new type was detected.

**Results:**

After six months, 26.3% had persistent infections, while 73.7% cleared the infection. After two years, 13.2% experienced persistence, and 86.8% cleared their infections. Among women who tested negative at baseline, 74 were re-tested and showed hr-HPV incidence of 4.05% after 24 months. Genotype persistence rates varied, with HPV68, 82, 53, 52, and 56 showing the highest persistence after six months. After 24 months, HPV59, 68, 66, 52, and 16 had the highest persistence. Additionally, 29.9% of women at six months had abnormal cytology, including Atypical squamous cells of undetermined significance (ASCUS) and High grade squamous intraepithelial lesion (HSIL), which was 10.3% of those tested.

**Conclusion:**

The findings show that most hr-HPV infections among rural Ethiopian women cleared within two years, with varying persistence and clearance rates across HPV types. This emphasizes the need for regular monitoring and targeted prevention in high HPV prevalence populations.

## Introduction

Human papillomavirus (HPV) is a group of more than 200 related viruses, with high-risk types being the primary causative agents of cervical cancer and other anogenital malignancies.^1^ In 2022, an estimated 662,301 women worldwide were diagnosed with cervical cancer, and approximately 348,874 died of the disease. It is the most commonly diagnosed cancer in 23 countries and the leading cause of cancer-related deaths in 36 countries.^2^ The vast majority of these countries are in sub-Saharan Africa, Melanesia, South America, and Southeast Asia.^3^ Cervical cancer is the second most common cancer in Ethiopia, with 8,168 new cases annually and approximately 5,975 deaths each year.^2^

Approximately 99.7% of cervical cancers are caused by persistent infection with hr-HPV genotypes.^4^ However, most HPV infections are transient, with many individuals clearing the virus within 1–2 years due to the immune response. More than half of HPV infections resolve within 6 months, and up to 90% are spontaneously cleared within 24 months.^5–7^ However, some high-risk types can evade immune detection and establish persistent infections, significantly increasing the risk of malignant progression. Additionally, reinfection with a new or the same HPV type may occur through a new partner, reinfection from the same partner, or reactivation of the initial infection.^8^ Studies indicate that only a small fraction of cervical intraepithelial neoplasia (CIN) grade II lesions progress to invasion, with about 22% advancing to carcinoma in situ. Furthermore, it is estimated that fewer than 50% of women with CIN3 develop invasive cervical cancer within 30 years.^9^

Infection of the cervical epithelium with carcinogenic HPV genotypes is the first step in the development of cervical cancer. However, hr-HPV infection alone is not sufficient for cancer to develop. The persistence of the infection is a crucial step in the process.^10^,^11^ Understanding the persistence, clearance, and reinfection rates of hr-HPV types over time is critical in identifying individuals at greater risk of progression to precancerous and cancerous lesions. It could also serve as a useful screening marker for cervical cancer. For example, a follow-up study by Iacobone et al^12^ confirmed that persistence of the same HPV genotype increases the odds of developing CIN2+ recurrence by 30-fold. Monitoring these dynamics provides insight into the natural history of HPV and informs public health strategies, such as targeted screening programs and vaccination efforts, to prevent persistent infections that lead to cancer.

Despite the lack of consensus, persistent infection is commonly defined as “finding the same genotype positive in two consecutive HPV tests.” However, the screening interval for type-specific infection varies widely across different reports.^13^,^14^ The time interval between two measurements significantly impacts persistence estimates because many infections are cleared within 2 years.^15^

The factors influencing whether an HPV infection will persist or clear are complex and include a range of host, viral, and environmental factors. Despite conflicting findings across studies, multiple-type infections have been identified as important cofactors in the persistence of high-risk infections.^16–18^ Regardless of the time frame, the primary determinants of HPV persistence are the specific HPV type and viral load at initial detection.^19^ Although the mechanisms regulating the persistence and progression of hr-HPV infections are not yet fully understood, certain environmental and exogenous factors have been identified as modifiers of the natural history of HPV infections leading to cervical cancer. Among these, coexisting sexually transmitted infections, high parity, smoking, and long-term use of oral contraceptives have been implicated as cofactors in HPV persistence.^20^,^21^ Additionally, reinfection with the same high-risk genotype or new high-risk genotypes can further exacerbate this risk.^22^

The incidence, genotype frequency, and prevalence of HPV infection vary across geographic regions. In Ethiopia, the population-based prevalence of hr-HPV infection is approximately 20%, with the most prevalent genotypes being HPV 16, 35, 52, 31, and 45.^23^ A systematic review of 3633 Ethiopian women with different cervical abnormalities reported HPV16, HPV52, HPV35, HPV18, and HPV56 as the most prevalent high-risk genotypes with 77.5% overall proportion of hr-HPV.^24^ In comparison, neighboring countries report varying HPV prevalence: 16.8%–41.6% in Kenya and 15.2%–73.2% in Uganda among women with normal cervical cytology.^25^ However, there is limited knowledge about the persistence and clearance of HPV infections specific to geographic regions and populations.^26–29^ Given its substantial public health impact, understanding the natural course of HPV infection—including persistence, clearance, and reinfection patterns—is crucial for developing effective prevention, screening, and treatment strategies. Few studies have investigated the persistence and clearance of hr-HPV infections in low- and middle-income countries, and none have been conducted in Ethiopia.

In Ethiopia, the overall coverage of cervical cancer screening remained very low despite the introduction of national VIA based screening program in 2015.^30^ This has been contributed due to the reliance on VIA that is with low uptake and requires well-trained providers, and performance varies widely.^31^ The other reason for this is due to limited access for HPV testing which is more accurate and high acceptance by rural women.^32^ Limited Infrastructure and Resources, Poor Awareness and Sociocultural Barriers, Shortage of Trained Health Workforce and Inadequate Follow-up and Treatment can also contribute to the limited coverage of cervical cancer screening in Ethiopia.^32^,^33^

There is ongoing uncertainty about whether HPV infections clear completely or become latent, with the potential for reactivation later in life. While reinfections are acknowledged, the factors contributing to reinfection remain poorly understood. This study aims to address gaps in current research by examining the dynamics of hr-HPV infection over a 2-year period, focusing on persistence, clearance, and reinfection rates, and associating them with clinical outcomes.

## Materials and Methods

### Study Design and Population

This follow-up study was part of a larger study conducted at the Butajira Health and Demographic Surveillance Site of Addis Ababa University, Ethiopia. The design and protocol of the larger study have been described in detail previously.^23^,^31^ The broader study began in January 2018 with the aim of investigating the role of self-sampled HPV DNA testing in improving cervical cancer screening uptake and determining the circulating HPV genotypes among unscreened and unvaccinated women in rural communities. Additionally, it sought to follow the same population over time to evaluate the persistence, clearance, and reinfection rates of HPV genotypes. In total, 1,020 women aged 30 to 49 years were invited for baseline screening, and 893 provided self-collected samples for HPV DNA testing. The exclusion criteria were pregnancy, refusal to participate, and being in active menses during sample collection.

### Follow‐Up Testing

Women eligible for this follow-up study included those who tested positive for hr-HPV at baseline (n = 157) and a randomly selected control group of women who tested negative (n = 80). To determine persistence, clearance, and reinfection rates, all 157 hr-HPV-positive women were invited to attend two follow-up visits for HPV DNA testing at 6 and 24 months. For follow-up, the women were contacted by local healthcare providers (health extension workers in the Ethiopian context) and invited to a district hospital in Butajira City. A physician collected samples from each participant for HPV testing at the hospital. Additionally, the women underwent a gynecological examination using VIA after sample collection. If the VIA result was positive, treatment was provided using cryotherapy. The gynecological examinations, including VIA and physician-collected samples, were performed by experienced gynecologists. During the 6-month follow-up visit, an additional sample was collected for cytological examination using a Papanicolaou (Pap) smear.

Sampling during the 24-month follow-up was conducted by inviting all baseline hr-HPV-positive women (n = 157) to the nearest health posts, where self-collected specimens were obtained for HPV DNA testing. To effectively detect precancerous lesions, all women who tested positive for hr-HPV infections—either due to type-specific persistence or reinfection with a new hr-HPV genotype—were invited to undergo colposcopy in Addis Ababa (approximately 150 km from the study area). Additionally, during the 24-month follow-up, approximately 13% of the women who tested hr-HPV negative at baseline (n = 80) were randomly selected and invited for HPV DNA testing as controls. This allowed for a comparison of type-specific hr-HPV reinfection rates between the baseline hr-HPV-positive and hr-HPV-negative groups. Women in the control group who were found to have new infections were retested 24 months after their baseline test.

**Figure 1:**
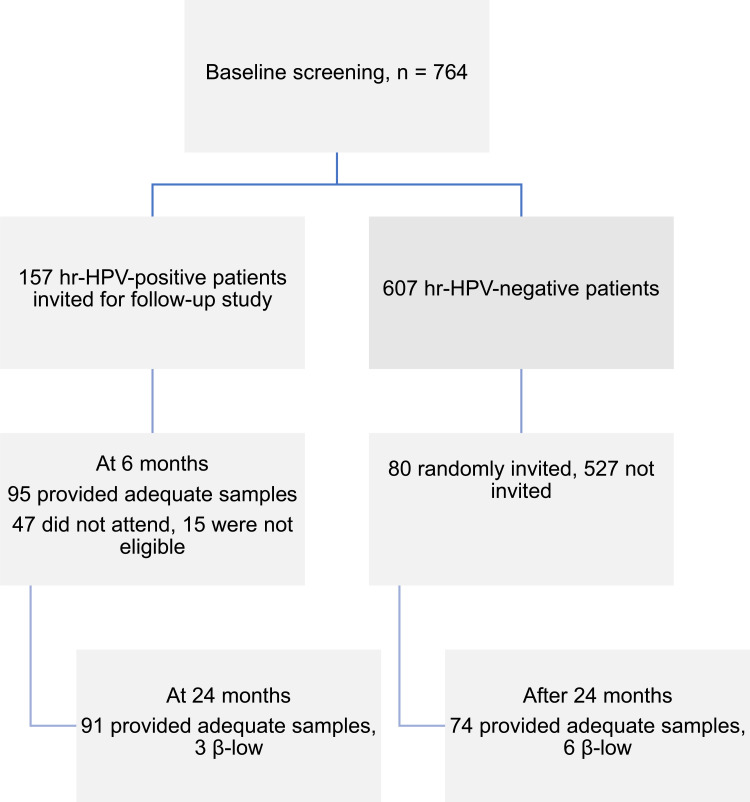
Follow-up study flow chart.

**Abbreviations**: β, beta-globin (a housekeeping gene used as an internal control); β-low, inadequate DNA content for HPV analysis according to our quality criteria; hr-HPV, high-risk human papillomavirus.

During follow-up visits at both time points, HPV genotype detection, cervical examination using VIA, cytology, colposcopy, and histological examination (if indicated) were performed. Changes in the hr-HPV infection status, clearance of hr-HPV infection, reinfection with a new hr-HPV type, and cytological and histological changes were documented. The study flow chart is shown in Figure 1.

### Sample Collection and HPV DNA Extraction

This study included three sampling points: baseline, 6 months, and 24 months. During the baseline and 24-month follow-up sampling, cervicovaginal samples were collected using a self-collection device (Evalyn Brush; Rovers Medical Devices, Oss, The Netherlands). The detailed protocol has been described previously.^23^ At the 6-month follow-up, physician-collected samples were obtained using a dedicated cervical cell collection device (Cervex-Brush; Rovers Medical Devices), a green, broom-like, soft, flexible brush designed for dual collection of ectocervical and endocervical cells in a single movement. The details of this procedure have also been described previously.^23^

The Evalyn Brush was processed as previously described.^23^ Briefly, the brush was removed from its plastic bag, and the tip was detached and placed into a 2-mL Eppendorf tube. It was then soaked in 1 mL phosphate-buffered saline overnight to release the cells from the dry Evalyn Brush. After centrifugation at 2,500 rpm for 5 minutes and vigorous vortexing for 1 minute, a 200-μL aliquot of the fluid was used for DNA extraction. For the ThinPrep-collected sample (Cervex-Brush), the sample was mixed thoroughly to ensure homogeneity, and 1 mL was taken and centrifuged at 14,000 rpm for 5 minutes. After discarding the supernatant, the pellet was resuspended in 200 μL of phosphate-buffered saline for extraction. DNA extraction was then performed using the Qiagen DNA Extraction Kit (QIAGEN, Hilden, Germany).

### Defining Persistence, Clearance, and Re-Infection

In our study, persistence, clearance, and reinfection of HPV were assessed for both overall hr-HPV infections and type-specific infections. Total hr-HPV persistence was defined as hr-HPV positivity at follow-up visits in women who were hr-HPV positive at baseline, regardless of genotype similarity. Similarly, total hr-HPV clearance was defined as a woman testing negative for any hr-HPV at follow-up visits after having tested positive for any hr-HPV at baseline.

Type-specific persistence was defined as the detection of the same HPV genotype at both baseline and follow-up using the same HPV DNA detection method. Likewise, type-specific clearance was defined as the proportion of women who were initially hr-HPV positive but no longer had the same hr-HPV type at follow-up. In other words, a change from a specific hr-HPV genotype positive status to negative was considered clearance. Reinfection was defined as the acquisition of a new hr-HPV genotype (other than the HPV genotype detected at baseline) after clearance of the initial infection.

### HPV Genotyping

In all rounds of testing, HPV genotyping was performed using BSGP5+/6+ polymerase chain reaction (PCR), followed by Luminex-MPG read-out, according to the protocol established by Schmitt et al in 2008. This assay detects both high-risk and low-risk HPV genotypes, including 6, 11, 16, 18, 26, 31, 33, 35, 39, 42, 43, 45, 51, 52, 53, 54, 56, 57, 58, 59, 66, 68a, 68b, 70, 72, 73, 82, and 90. The genotyping was conducted at Charité Universitätsmedizin Berlin, Germany. Details of the methods have been described previously.^23^

### Cytology

In our study, Pap smear slides were prepared and stained by trained laboratory professionals, and a senior pathologist performed the reading and interpretation to determine the stage of cervical cell abnormalities based on the Bethesda 2014 protocol.

## Results

### Baseline and Follow Up Characteristics of Study Participants

In this study, we followed women aged 30–49 years who tested hr-HPV positive during the initial screening, along with a randomly selected control group of women who tested negative for any hr-HPV genotype. Among the baseline hr-HPV-positive women (n = 157), 110 (70%) attended the 6-month follow-up, with 95 (60.5%) providing cervical samples for HPV DNA testing, while 15 (9.6%) were excluded because of pregnancy or active menses at the time of sample collection. At the 24-month follow-up, 94 (59.9%) of the hr-HPV-positive women participated in sample collection. Consequently, the study recorded a loss to follow-up of 47 (30.0%) participants at the 6-month visit and 63 (40.1%) participants at the 24-month visit.

Among the 94 samples collected at the 24-month follow-up, 91 (96.8%) had adequate cellularity for HPV DNA detection, while the remaining 3 (3.2%) were inadequate because of low internal control values for β-globin PCR. A total of 70 (44.6%) women completed both the 6- and 24-month follow-up sample collections. The primary reasons for loss to follow-up were lack of spousal support, women relocating after the initial screening, conflicts in the study area during follow-up data collection, reluctance to repeat tests because of feeling healthy, and pregnancy.

### Prevalence of HPV Infections

The hr-HPV prevalence at baseline screening was 157 (20.5%). Among the women who were hr-HPV positive at baseline and attended the 6- and 24-month follow-ups, 25 (26.3%) and 14 (15.4%) tested positive for any HPV type, respectively. Additionally, 25 (26.3%) and 12 (13.2%) of these women remained hr-HPV positive after 6 and 24 months, respectively (Table 1). Among the 607 hr-HPV-negative women at baseline, 80 (13.0%) were randomly selected as controls for the follow-up study. Of these, 74 (92.5%) had adequate cellularity for HPV detection, while 6 (7.5%) had inadequate cellularity because of low internal control values for β-globin PCR. After 24 months of follow-up testing, only 3 (4.05%) women in the control group tested positive for hr-HPV, while 71 (95.9%) remained hr-HPV negative.

**Table 1:** HPV Positivity Rates at Baseline and at 6- and 24-Month Follow-Up Screenings in Butajira, Ethiopia

HPV Infection	Baseline Screening (Tested Women=764) (n, %)	At 6 Months, from the Baseline hr-HPV Positives, (Tested Women =95) (n, %)	At 24 Months, from the Baseline hr-HPV Positives (Tested Women = 91) (n, %)
Any HPV	177 (23.2)	25 (26.3)	14 (15.4)
hr-HPV	157 (20.5)	25 (26.3)	12 (13.2)
lr-HPV	79 (10.3)	5 (5.3)	4 (4.4)

### hr-HPV Type-Specific Point Prevalences

The type-specific prevalence of HPV genotypes was assessed separately at each follow-up visit. At the baseline screening, 18 hr-HPV genotypes were detected (16, 18, 26, 31, 33, 35, 39, 45, 51, 52, 53, 56, 58, 59, 66, 68, 73, and 82). At the 6-month follow-up, 12 hr-HPV genotypes were identified (16, 18, 31, 35, 39, 51, 52, 53, 56, 66, 68, and 82), while at the 24-month follow-up, 9 hr-HPV genotypes were detected (16, 31, 39, 52, 56, 59, 66, 68, and 82).

Despite HPV16 being the most frequent genotype across all time points, HPV52 after 6 months and HPVs 52, 66, and 59 after 24 months were also among the most prevalent genotypes (Table 2). Among the women who underwent HPV DNA testing at 6 months (n = 95), HPV16 and HPV52 were equally frequent (28%), followed by HPV82 (24%), HPV35 (16%), HPV53 (16%), and HPV68 (12%). At the 24-month follow-up (n = 91), HPV16, HPV52, HPV66, and HPV59 were equally frequent (25.0%), followed by HPV56 (16.7%) (Table 2).

**Table 2:** Type-Specific HPV Point Prevalences During Follow-Up in Butajira, Ethiopia

		Follow-Up Number (%) of Cases	
HPV	Baseline n, %	6^th^ Months n, %	24^th^ Months n, %
hr-HPV positive	157 (20.5)	25 (26.3)	12 (13.2)
hr-HPV negative	607 (79.5)	70 (73.7)	79 (86.8)
**HPV genotype among the hr-HPV positive women**			
HPV 16	101 (64.3)	7 (28)	3 (25)
HPV 18	16 (10.2)	1 (4)	0
HPV 26	12 (7.6)	0	0
HPV 31	25 (15.9)	1(4)	1(8.3)
HPV 33	3 (1.9)	0	0
HPV 35	36 (22.9)	4 (16)	0
HPV 39	6 (3.8)	1(4)	1(8.3)
HPV 45	17 (10.8)	0	0
HPV 51	13 (8.3)	2 (8)	0
HPV 52	28 (17.8)	7 (28)	3 (25)
HPV 53	10 (6.4)	4 (16)	0
HPV 56	10 (6.4)	2 (8)	2 (16.7)
HPV 58	5 (3.2)	0	0
HPV 59	4 (2.5)	0	3 (25)
HPV 66	8 (5.1)	1(4)	3 (25)
HPV 68	4 (2.5)	3 (12)	1 (8.3)
HPV 73	1 (0.6)	0	0
HPV 82	9 (5.7)	6 (24)	1(8.3)

### Genotype-Specific hr-HPV Persistence and Clearance During Follow-Up

At the baseline screening, 177 (23.2%) of the 764 participants tested HPV positive. After 6 months, 95 of these women participated in follow-up testing, with 70 (73.7%) clearing their infections and 25 (26.3%) remaining hr-HPV positive. Among the 25 hr-HPV infections detected at 6 months, 9 (36%) were new infections, while 16 (64%) were genotype-specific persistent infections. At the 24-month follow-up, among the 91 women tested, 77 (84.6%) cleared their infections, while 14 (15.4%) had persistent infections with any HPV type.

Among the hr-HPV-positive women at the 6-month follow-up, HPV68, 82, 53, 52, and 56 were the most persistent genotypes, with persistence rates of 100%, 75%, 42.9%, 31%, and 25%, respectively. By contrast, HPV18, 26, 33, 39, 45, 51, 58, and 59 had 100% clearance rates, making them the least persistent genotypes at 6 months. The persistence rate of HPV16 among the study participants was 11.3% (Table 3). At the 24-month follow-up, HPV59, 68, 66, 52, and 16 persisted at rates of 50.0%, 50.0%, 20.0%, 15.8%, and 3.5%, respectively (Table 3).

**Table 3:** Type-Specific Persistence/Clearance/New Infection of hr-HPV at 6 and 24 Months of Follow-Up

			hr-HPV Infection Dynamics During the Follow up									
	6^th^ Month	24^th^ Month	Persistence				Clearance				New Infection	
HPV Type	Prevalence at Baseline from the follow up attended women (n=95)	Prevalence at Baseline from the follow up attended women (n=91)	6^th^ Month		24^th^ Month		6^th^ Month		24^th^ Month		6^th^ Month	24^th^ Month
			n	%	n	%	n	%	n	%	n	n
HPV 16	62	57	7	11.3	2	3.5	55	88.7	55	96.5	0	0
HPV 18	12	13	0	0.0	0	0.0	12	100.0	13	100.0	1	0
HPV 26	10	10	0	0.0	0	0.0	10	100.0	10	100.0	0	0
HPV 31	12	12	1	8.3	0	0.0	11	91.7	12	100.0	0	1
HPV 33	2	3	0	0.0	0	0.0	2	100.0	3	100.0	0	0
HPV 35	24	21	3	12.5	0	0.0	21	87.5	21	100.0	1	0
HPV 39	6	4	0	0.0	0	0.0	6	100.0	4	100.0	1	1
HPV 45	13	14	0	0.0	0	0.0	13	100.0	14	100.0	0	0
HPV 51	7	10	0	0.0	0	0.0	7	100.0	10	100.0	2	0
HPV 52	19	19	6	31.6	3	15.8	13	68.4	16	84.2	1	0
HPV 53	7	6	3	42.9	0	0.0	4	57.1	6	100.0	1	0
HPV 56	8	7	2	25.0	0	0.0	6	75.0	7	100.0	0	2
HPV 58	3	5	0	0.0	0	0.0	3	100.0	5	100.0	0	0
HPV 59	3	2	0	0.0	1	50.0	3	100.0	1	50.0	0	2
HPV 66	7	5	1	14.3	1	20.0	6	85.7	4	80.0	0	2
HPV 68	2	2	2	100.0	1	50.0	0	0.0	1	50.0	1	0
HPV 82	4	4	3	75.0	0	0.0	1	25.0	4	100.0	3	1

### Cervical Neoplasia in Relation to hr-HPV Infection

At the 6-month follow-up, cervical cytology using Pap smears was performed for 97 women. Among them, 10.3% were diagnosed with a HSIL, followed by 9.3% with ASCUS) and 8.2% with a low-grade squamous intraepithelial lesion (LSIL) (Table 4).

**Table 4:** Pap Smear Test Results in the Butajira Follow-Up Study

Cytology	n	%
NILM	59	60.8
Inadequate	9	9.3
ASCUS	9	9.3
ASC-H	2	2.1
LSIL	8	8.2
HSIL	10	10.3
Total	97	100

**Abbreviations**: HSIL, High grade squamous intraepithelial lesion; ASC-H, Atypical squamous cells—cannot exclude HSIL; LSIL, Low-grade squamous intraepithelial lesion; ASCUS, Atypical squamous cells of undetermined significance; NILM, Negative for Intraepithelial Lesion or Malignancy; inadequate, sample was inadequate for cytological examination.

**Figure 2:**
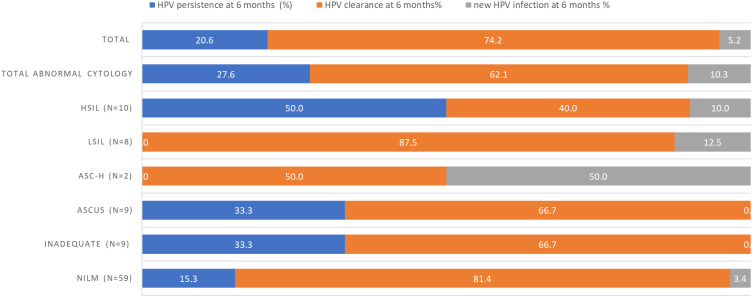
Persistence and clearance of hr-HPV infections in relation to evidence of cervical cytological results at 6-month follow-up.

Figure 2 below illustrates the course of HPV infection in relation to cytological results at follow-up. Among the 29 individuals with abnormal cytology, including ASCUS at the 6-month follow-up, 8 (27.6%) had type-specific persistent hr-HPV infections, while 18 (62.1%) cleared their baseline hr-HPV infections. Additionally, 3 (10.3%) of the cytologically abnormal cases had new hr-HPV infections different from their baseline infection.

Of the 10 women with HSIL at the 6-month follow-up, 5 (50%) had persistent type-specific hr-HPV infections, while 4 (40%) cleared their hr-HPV infections, and 1 (10%) acquired a new HPV infection. By contrast, among the 8 women with LSIL, 7 (87.5%) cleared their baseline hr-HPV infections, while 1 (12.5%) acquired a new hr-HPV infection different from the baseline (Figure 2). Furthermore, at the 6-month follow-up, 9 (15.3%) of the women negative for an intraepithelial lesion or malignancy had type-specific hr-HPV infections.

## Discussion

This study provides essential data on the longitudinal dynamics of HPV infection at the population level in south central Ethiopia. This information is particularly valuable given the strong association between cervical cancer and persistent hr-HPV genotypes and the lack of similar data in this population, despite the high prevalence of HPV infection and cervical cancer in Ethiopia.^3^,^23^ Furthermore, our study is the first to report on the natural history of hr-HPV infection among a rural, unscreened population in Ethiopia. The baseline prevalence of HPV infection was 23.2% for any HPV and 20.5% for hr-HPV, marking the first determination of circulating genotypes at the population level. The study aimed to follow all hr-HPV-positive women for 2 years, with HPV testing conducted at 6 and 24 months. Approximately 60% of the invited women attended their follow-up visits, and the prevalence of hr-HPV among these women declined to 26.3% at 6 months and 13.2% at 24 months, indicating high HPV clearance rates within the population.

Most of the hr-HPV infections cleared during our follow-up evaluation, with clearance rates of 73.3% at 6 months and 86.8% at 24 months. Our findings align with previous studies of Korean women,^34^ which reported that up to 90% of hr-HPV infections clear within 2 years.^11^,^35^,^36^ However, HPV viral clearance rates vary across countries due to multiple factors. Our results also support the observation that HPV clearance is most frequent within the first 6 months of follow-up.^37^

The natural history of HPV infection is influenced by various environmental, host, and viral factors. Understanding population-specific factors that contribute to the clearance and persistence of HPV infections is crucial because hr-HPV persistence for 1 or 2 years is a strong predictor of cervical precancer and cancer.^19^,^38^ In our follow-up study, new hr-HPV genotype acquisitions were observed after clearance. While 70 women cleared their infections at 6 months and 77 at 24 months, 12.9% and 7.8% of them, respectively, acquired a new HPV infection, as indicated by the detection of a different genotype from baseline. This suggests that acquiring a new HPV infection after clearance is more common than acquiring an infection after a previously negative HPV test. Our findings support this: among the 74 women who were hr-HPV negative at baseline, only 3 (4.05%) acquired an hr-HPV infection during follow-up. Importantly, while new infections are common, persistent infections pose a significantly higher risk of malignant transformation compared to those with a type change after clearance.^39^

However, HPV persistence varies depending on the target population and immune status of the women. At 24 months, our study reported a relatively lower persistence rate (13.2%) than in some previous studies, while 26.3% of women had persistent infections at 6 months. By contrast, the 2-year persistence rates reported in other studies include 19.2% in Brazil,^40^ 49.1% in Italy,^41^ 31.4%^42^ and 26.9%^29^ in Denmark, 39.0% in the United States,^43^ and 44.1% in the Netherlands.^44^ These differences may be attributed to variations in the target populations, differences in risk factors for persistent infection, and testing intervals. Additional cofactors influencing HPV persistence include smoking and hormonal exposure, and because the smoking rates were very low in our study population,^45^,^46^ this may explain the low persistence rate observed. Another possible factor contributing to the high clearance rate in our study is the age of the women, with a mean age of 33 years, as studies suggest that younger women have a higher capacity for HPV clearance.^36^ Furthermore, the lack of a standardized definition of HPV persistence across studies contributes to variations in reported persistence rates. The most commonly used definition considers HPV persistence as the detection of the same HPV type at two or more time points during follow-up,^47^ while some studies require persistence to be confirmed at three consecutive visits spaced 2 to 24 months apart. A few studies define persistence based on the proportion of visits that are HPV-positive throughout the study duration, offering a broader perspective on how often an individual tests positive over time.^48^ Other studies focus on the duration until clearance, defining persistence as the period during which an HPV type remains detectable before a negative result is obtained.^49^ The diversity in definitions highlights the need for standardized criteria in future research to enable more reliable comparisons and conclusions regarding HPV persistence and its implications for public health strategies.

Previous studies have shown that different oncogenic HPV types vary in their clearance duration and carcinogenic potential. In our study, the most persistent HPV type at 6 months was HPV 68 (100%), followed by HPV 82 (75.0%) and HPV 53 (42.9%). At 2 years, the most persistent genotypes were HPV 68 and HPV 59 (both 50.0%), followed by HPV 66 (20.0%) and HPV 52 (15.8%). However, because there were only two cases of HPV 68 and HPV 59 infections, comparing type-specific persistence and clearance is challenging due to the small sample size. Previous studies have identified HPV 16 and 18 as the most persistent genotypes.^40–42^ Most research on female genital HPV infections has consistently reported HPV 16 as the most persistent genotype. Additionally, a 6-year follow-up study from the Finnish Family HPV Study found that HPV 16 remained the most common genotype throughout the entire study period.^26^

Another key aim of this study was to assess the magnitude of abnormal cytological findings among women with persistent hr-HPV infections because persistent hr-HPV infection is the single strongest predictor of cervical cancer development.^50^ In our study, the prevalence of abnormal cytology, including ASCUS, was 30% among the 97 women who attended the 6-month follow-up. This finding aligns with previous studies that examined the prevalence of hr-HPV in women with abnormal cervical cytology. For instance, Song et al reported that the overall prevalence of hr-HPV in women with abnormal cervical cytology was 32%.^51^

Studies have consistently shown that women with persistent hr-HPV infections are significantly more likely to develop cervical neoplasia than are women who clear their infections.^52–54^ Similarly, in our study, women with persistent hr-HPV had a higher likelihood of neoplasia than those without hr-HPV. However, other factors may also contribute to the development of neoplasia, such as infections with highly virulent, high-copy-number hr-HPV that persist for a shorter duration but are not classified as persistent under our time point definitions. Notably, among the 10 women with HSIL, 50% had persistent type-specific hr-HPV infections. The high rate of previous hr-HPV clearance among women with abnormal cytology in our study could be attributed to differences in cytology quality, HPV sampling methods, and sample size across studies. Additionally, age differences between our study population and those in other studies may also influence clearance rates because HPV clearance is known to vary by age group.

## Conclusion

This study provided valuable insights into changes in hr-HPV status over 6 months and 2 years among Ethiopian women with an initial positive hr-HPV test. We found that most HPV infections resolved spontaneously including genotypes 18, 26, 33, 39, 45, 51 and 58 while small fraction of women specifically with HPV 16, 52, 66 and 68 experience persistent infection or acquiring new HPV infections. The persisting hr-HPV genotypes varied considerably, with no single dominant type observed. These findings underscore the importance of re-inviting and closely following women who test hr-HPV positive to identify those at risk of developing cervical lesions. Further large-scale studies are needed to better understand the natural history of specific HPV genotypes and the factors influencing their persistence in Ethiopia.

## References

[1] Okunade KS. Human papillomavirus and cervical cancer. *J Obstet Gynaecol J Inst Obstet Gynaecol*. 2020;40(5):602–608. doi:10.1080/01443615.2019.1634030 PMC7062568 31500479

[2] Ferlay J, Colombet M, Soerjomataram I, et al. Cancer statistics for the year 2020: an overview. *Int, J, Cancer*. 2021;149(4):778–789. doi:10.1002/ijc.33588 33818764

[3] Sung H, Ferlay J, Siegel RL, et al. Global cancer statistics 2020: GLOBOCAN estimates of incidence and mortality worldwide for 36 cancers in 185 countries. *CA Cancer J Clin*. 2021;71(3):209–249. doi:10.3322/caac.21660 33538338

[4] Walboomers JM, Jacobs MV, Manos MM, et al. Human papillomavirus is a necessary cause of invasive cervical cancer worldwide. *J Pathol*. 1999;189(1):12–19. doi:10.1002/(SICI)1096-9896(199909)189:1<12::AID-PATH431>3.0.CO;2-F 10451482

[5] Rodriguez AC, Burk R, Herrero R, et al. The natural history of human papillomavirus infection and cervical intraepithelial neoplasia among young women in the Guanacaste cohort shortly after initiation of sexual life. *Sex Transm Dis*. 2007;34(7):494–502. doi:10.1097/01.olq.0000251241.03088.a0 17237737

[6] Oh JK, Ju YH, Franceschi S, Quint W, Shin HR. Acquisition of new infection and clearance of type-specific human papillomavirus infections in female students in Busan, South Korea: a follow-up study. *BMC Infect Dis*. 2008;8:1–6. doi:10.1186/1471-2334-8-13 18234114 PMC2257960

[7] Castle PE, Schiffman M, Wheeler CM, Solomon D. Evidence for frequent regression of cervical intraepithelial neoplasia-grade 2. *Obstet Gynecol*. 2009;113(1):18–25. doi:10.1097/AOG.0b013e31818f5008 19104355 PMC2694845

[8] Richardson H, Kelsall G, Tellier P, et al. The natural history of type-specific human papillomavirus infections in female university students. *Cancer Epidemiol Biomarkers Prev*. 2003;12(6):485–490. 12814991

[9] McCredie MR, Sharples KJ, Paul C, et al. Natural history of cervical neoplasia and risk of invasive cancer in women with cervical intraepithelial neoplasia 3: a retrospective cohort study. *Lancet Oncol*. 2008;9(5):425–434. doi:10.1016/S1470-2045(08)70103-7 18407790

[10] Schiffman M, Wentzensen N, Wacholder S, Kinney W, Gage JC, Castle PE. Human papillomavirus testing in the prevention of cervical cancer. *J Natl Cancer Inst*. 2011;103(5):368–383. doi:10.1093/jnci/djq562 21282563 PMC3046952

[11] Gravitt PE. The known unknowns of HPV natural history. *J Clin Invest*. 2011;121(12):4593–4599. doi:10.1172/JCI57149 22133884 PMC3225991

[12] Iacobone AD, Radice D, Sandri MT, et al. Human papillomavirus same genotype persistence and risk of cervical intraepithelial neoplasia 2+ recurrence. *Cancers*. 2021;13(15):1–12. doi:10.3390/cancers13153664 PMC8345074 34359566

[13] Rositch AF, Koshiol J, Hudgens MG, et al. Patterns of persistent genital human papillomavirus infection among women worldwide: a literature review and meta-analysis. *Int J Cancer*. 2013;133(6):1271–1285. doi:10.1002/ijc.27828 22961444 PMC3707974

[14] Mũoz N, Hernandez-Suarez G, Méndez F, et al. Persistence of HPV infection and risk of high-grade cervical intraepithelial neoplasia in a cohort of Colombian women. *Br J Cancer*. 2009;100(7):1184–1190. doi:10.1038/sj.bjc.6604972 19293802 PMC2669994

[15] Marks MA, Castle PE, Schiffman M, Gravitt PE. Evaluation of any or type-specific persistence of high-risk human papillomavirus for detecting cervical precancer. *J Clin Microbiol*. 2012;50(2):300–306. doi:10.1128/JCM.05979-11 22162556 PMC3264159

[16] Trottier H, Mahmud S, Prado JCM, et al. Type-specific duration of human papillomavirus infection: implications for human papillomavirus screening and vaccination. *J Infect Dis*. 2008;197(10):1436–1447. doi:10.1086/587698 18419547 PMC7889327

[17] Kulmala SMA, Shabalova IP, Petrovitchev N, et al. Type-specific persistence of high-risk human papillomavirus infections in the new independent states of the former Soviet Union cohort study. *Cancer Epidemiol Biomarkers Prev*. 2007;16(1):17–22. doi:10.1158/1055-9965.EPI-06-0649 17220327

[18] Campos NG, Rodriguez AC, Castle PE, et al. Persistence of concurrent infections with multiple human papillomavirus types: a population-based Cohort Study. *J Infect Dis*. 2011;203(6):823–827. doi:10.1093/infdis/jiq131 21257737 PMC3071138

[19] Castle PE, Rodríguez AC, Burk RD, et al. Long-term persistence of prevalently detected human papillomavirus infections in the absence of detectable cervical precancer and cancer. *J Infect Dis*. 2011;203(6):814–822. doi:10.1093/infdis/jiq116 21343148 PMC3071125

[20] Jensen KE, Schmiedel S, Norrild B, Frederiksen K, Iftner T, Kjaer SK. Parity as a cofactor for high-grade cervical disease among women with persistent human papillomavirus infection: a 13-year follow-up. *Br J Cancer*. 2013;108(1):234–239. doi:10.1038/bjc.2012.513 23169283 PMC3553518

[21] Yetimalar H, Kasap B, Cukurova K, Yildiz A, Keklik A, Soylu F. Cofactors in human papillomavirus infection and cervical carcinogenesis. *Arch Gynecol Obstet*. 2012;285(3):805–810. doi:10.1007/s00404-011-2034-3 21830008

[22] Louvanto K, Rautava J, Willberg J, et al. Genotype-specific incidence and clearance of human papillomavirus in oral mucosa of women: a six-year follow-up study. *PLoS One*. 2013;8(1):e53413. doi:10.1371/journal.pone.0053413 23301068 PMC3536668

[23] Teka B, Gizaw M, Ruddies F, et al. Population-based human papillomavirus infection and genotype distribution among women in rural areas of South Central Ethiopia. *Int, J, Cancer*. 2021;148(3):723–730. doi:10.1002/ijc.33278 32875552

[24] Derbie A, Mekonnen D, Nibret E, Maier M, Woldeamanuel Y, Abebe T. Human papillomavirus genotype distribution in Ethiopia: an updated systematic review. *Virol J*. 2022;19(1). doi:10.1186/s12985-022-01741-1 PMC8760777 35033141

[25] Bruni L, Albero G, Serrano B, et al. Human papillomavirus and related diseases report. 2021.

[26] Louvanto K, Rintala MA, Syrjänen KJ, Grénman SE, Syrjänen SM. Genotype-specific persistence of genital human papillomavirus (HPV) infections in women followed for 6 years in the Finnish Family HPV study. *J Infect Dis*. 2010;202(3):436–444. doi:10.1086/653826 20557239

[27] Miranda PM, Silva NNT, Pitol BCV, et al. Persistence or clearance of human papillomavirus infections in women in Ouro Preto, Brazil. *Biomed Res Int*. 2013;2013:1–6. doi:10.1155/2013/578276 PMC3835752 24298551

[28] Schmeink CE, Massuger LFAG, Lenselink CH, et al. Prospective follow-up of 2,065 young unscreened women to study human papillomavirus incidence and clearance. *Int, J, Cancer*. 2013;133(1):172–181. doi:10.1002/ijc.27986 23233366

[29] Nielsen A, Kjaer SK, Munk C, Osler M, Iftner T. Persistence of high-risk human papillomavirus infection in a population-based cohort of Danish women. *J Med Virol*. 2010;82(4):616–623. doi:10.1002/jmv.21750 20166190

[30] Pillars S. NCCP ethiopia final submitted PDF_1.

[31] Gizaw M, Teka B, Ruddies F, et al. Uptake of cervical cancer screening in ethiopia by self-sampling HPV DNA compared to visual inspection with acetic acid: a cluster randomized trial. *Cancer Prev Res*. 2019;12(9):609–616. doi:10.1158/1940-6207.CAPR-19-0156 31337647

[32] Hussein K, Wafula F, Kassie GM, Kokwaro G. Barriers and facilitators to implementation of the Ethiopian national cancer control plan strategies: implications for cervical cancer services in Ethiopia. *PLOS Glob Public Heal*. 2024;4(7):1–15. doi:10.1371/journal.pgph.0003500 PMC11262691 39037972

[33] Mohamed ZK, Amare YW, Getahun MS, Negussie YM, Gurara AM. Cervical cancer screening service utilization and associated factors among women living with HIV receiving anti-retroviral therapy at Adama Hospital Medical College, Ethiopia. *SAGE Open Nurs*. 2023;9. doi:10.1177/23779608231152072 PMC9885028 36726790

[34] Ingabire C, Lim MK, Won YJ, Oh JK. Human papillomavirus genotype-specific persistence and potential risk factors among Korean women: results from a 2-year follow-up study. *Cancer Res Treat*. 2018;50(3):813–822. doi:10.4143/crt.2017.340 28814070 PMC6056951

[35] Bosch FX, Burchell AN, Schiffman M, et al. Epidemiology and natural history of human papillomavirus infections and type-specific implications in cervical neoplasia. *Vaccine*. 2008;26(SUPPL. 10):K1–K16. doi:10.1016/j.vaccine.2008.05.064 18847553

[36] Plummer M, Schiffman M, Castle PE, Maucort-Boulch D, Wheeler CM. A 2-year prospective study of human papillomavirus persistence among women with a cytological diagnosis of atypical squamous cells of undetermined significance or low-grade squamous intraepithelial lesion. *J Infect Dis*. 2007;195(11):1582–1589. doi:10.1086/516784 17471427

[37] de Sanjosé S, Brotons M, Pavón MA. The natural history of human papillomavirus infection. *Best Pract Res Clin Obstet Gynaecol*. 2018;47:2–13. doi:10.1016/j.bpobgyn.2017.08.015 28964706

[38] Kjær SK, Frederiksen K, Munk C, Iftner T. Long-term absolute risk of cervical intraepithelial neoplasia grade 3 or worse following human papillomavirus infection: role of persistence. *J Natl Cancer Inst*. 2010;102(19):1478–1488. doi:10.1093/jnci/djq356 20841605 PMC2950170

[39] Elfgren K, Elfström KM, Naucler P, Arnheim-Dahlström L, Dillner J. Management of women with human papillomavirus persistence: long-term follow-up of a randomized clinical trial. *Am J Obstet Gynecol*. 2017;216(3):264.e1–264.e7. doi:10.1016/j.ajog.2016.10.042 27825977

[40] Rosa MI, Fachel JMG, Rosa DD, Medeiros LR, Igansi CN, Bozzetti MC. Persistence and clearance of human papillomavirus infection: a prospective cohort study. *Am J Obstet Gynecol*. 2008;199(6):617.e1–617.e7. doi:10.1016/j.ajog.2008.06.033 18799155

[41] Sammarco ML, Del Riccio I, Tamburro M, Grasso GM, Ripabelli G. Type-specific persistence and associated risk factors of human papillomavirus infections in women living in central Italy. *Eur J Obstet Gynecol Reprod Biol*. 2013;168(2):222–226. doi:10.1016/j.ejogrb.2013.01.012 23395560

[42] Stensen S, Kjaer SK, Jensen SM, et al. Factors associated with type-specific persistence of high-risk human papillomavirus infection: a population-based study. *Int J Cancer*. 2016;138(2):361–368. doi:10.1002/ijc.29719 26238558

[43] Ralston Howe E, Li Z, McGlennen RC, Hellerstedt WL, Downs LS. Type-specific prevalence and persistence of human papillomavirus in women in the United States who are referred for typing as a component of cervical cancer screening. *Am J Obstet Gynecol*. 2009;200(3):245.e1–245.e7. doi:10.1016/j.ajog.2008.10.050 19254582

[44] Schmeink CE, Melchers WJG, Siebers AG, Quint WGV, Massuger LFAG, Bekkers RLM. Human papillomavirus persistence in young unscreened women, a prospective cohort study. *PLoS One*. 2011;6(11):1–8. doi:10.1371/journal.pone.0027937 PMC3223200 22132173

[45] Schoenmaker N, Hermanides J, Davey G. Prevalence and predictors of smoking in Butajira town, Ethiopia. *Ethiop J Heal Dev*. 2006;19(3). doi:10.4314/ejhd.v19i3.9996

[46] Wubegzier M, Alemayehu W. Determinants of low family planning use and high unmet need in Butajira District, South Central Ethiopia. *Reprod Health*. 2011;8(1):1–8. doi:10.1186/1742-4755-8-1 22151888 PMC3248357

[47] Koshiol J, Lindsay L, Pimenta JM, Poole C, Jenkins D, Smith JS. Persistent human papillomavirus infection and cervical neoplasia: a systematic review and meta-analysis. *Am J Epidemiol*. 2008;168(2):123–137. doi:10.1093/aje/kwn036 18483125 PMC2878094

[48] Sycuro LK, Long FX, Hughes JP, et al. Persistence of genital human papillomavirus infection in a long-term follow-up study of female university students. *J Infect Dis*. 2008;198(7):971–978. doi:10.1086/591625 18694334

[49] Zhao M, Kang P, Zhu L, et al. Global pattern of persistent human papillomavirus infection in female genital tract: an update system review and meta-analysis. *iScience*. 2024;27(10):110991. doi:10.1016/j.isci.2024.110991 39474077 PMC11519437

[50] Muñoz N, Bosch FX, de Sanjosé S, et al. Epidemiologic classification of human papillomavirus types associated with cervical cancer. *N Engl J Med*. 2003;348(6):518–527. doi:10.1056/nejmoa021641 12571259

[51] Song L, Lyu Y, Ding L, et al. Prevalence and genotype distribution of high-risk human papillomavirus infection in women with abnormal cervical cytology: a population-based study in Shanxi Province, China. *Cancer Manag Res*. 2020;12:12583–12591. doi:10.2147/CMAR.S269050 33324103 PMC7733379

[52] Cuschieri KS, Cubie HA, Whitley MW, et al. Persistent high risk HPV infection associated with development of cervical neoplasia in a prospective population study. *J Clin Pathol*. 2005;58(9):946–950. doi:10.1136/jcp.2004.022863 16126875 PMC1770812

[53] Wallin KL, Wiklund F, Angström T, et al. Type-specific persistence of human papillomavirus DNA before the development of invasive cervical cancer. *N Engl J Med*. 1999;341(22):1633–1638. doi:10.1056/NEJM199911253412201 10572150

[54] Kjaer SK, Van den Brule AJC, Paull G, et al. Type specific persistence of high risk human papillomavirus (HPV) as indicator of high grade cervical squamous intraepithelial lesions in young women: population based prospective follow up study. *Br Med J*. 2002;325(7364):572–576. doi:10.1136/bmj.325.7364.572 12228133 PMC124551

